# Coordination of cell division and chromosome segregation by iron and a sRNA in *Escherichia coli*

**DOI:** 10.3389/fmicb.2024.1493811

**Published:** 2024-10-25

**Authors:** Evelyne Ng Kwan Lim, Marc Grüll, Nadia Larabi, David Lalaouna, Eric Massé

**Affiliations:** Department of Biochemistry and Functional Genomics, RNA Group, Université de Sherbrooke, Sherbrooke, QC, Canada

**Keywords:** cell division, small RNAs, chromosome segregation, iron starvation, RyhB, ZapB

## Abstract

Iron is a vital metal ion frequently present as a cofactor in metabolic enzymes involved in central carbon metabolism, respiratory chain, and DNA synthesis. Notably, iron starvation was previously shown to inhibit cell division, although the mechanism underlying this observation remained obscure. In bacteria, the sRNA RyhB has been intensively characterized to regulate genes involved in iron metabolism during iron starvation. While using the screening tool MAPS for new RyhB targets, we found that the mRNA *zapB*, a factor coordinating chromosome segregation and cell division (cytokinesis), was significantly enriched in association with RyhB. To confirm the interaction between RyhB and *zapB* mRNA, we conducted both *in vitro* and *in vivo* experiments, which showed that RyhB represses *zapB* translation by binding at two distinct sites. Microscopy and flow cytometry assays revealed that, in the absence of RyhB, cells become shorter and display impaired chromosome segregation during iron starvation. We hypothesized that RyhB might suppress ZapB expression and reduce cell division during iron starvation. Moreover, we observed that deleting *zapB* gene completely rescued the slow growth phenotype observed in *ryhB* mutant during strict iron starvation. Altogether, these results suggest that during growth in the absence of iron, RyhB sRNA downregulates *zapB* mRNA, which leads to longer cells containing extra chromosomes, potentially to optimize survival. Thus, the RyhB-*zapB* interaction demonstrates intricate regulatory mechanisms between cell division and chromosome segregation depending on iron availability in *E. coli*.

## Introduction

DNA replication and cell division are fundamental processes that are essential for the survival and proliferation of all cells. Successful cell division and chromosome replication require the careful coordination of multiple factors, including accurate DNA replication, precise regulation of the cell cycle, and proper segregation of chromosomes to the daughter cells. Bacterial DNA replication is a tightly regulated process that involves the initiation and progression of the replication fork, the elongation of the nascent DNA strands, and the termination of DNA synthesis, which are coordinated by a network of proteins and regulatory factors (reviewed in Reyes-Lamothe et al., 2012). The process of bacterial cell division, or cytokinesis, requires the formation of a cell wall and the generation of a septum at the midcell. The division septum then acts as a scaffold for the recruitment of the protein complex required for cell division, also known as the divisome (reviewed in Mahone and Goley, 2020).

**Figure 1:**
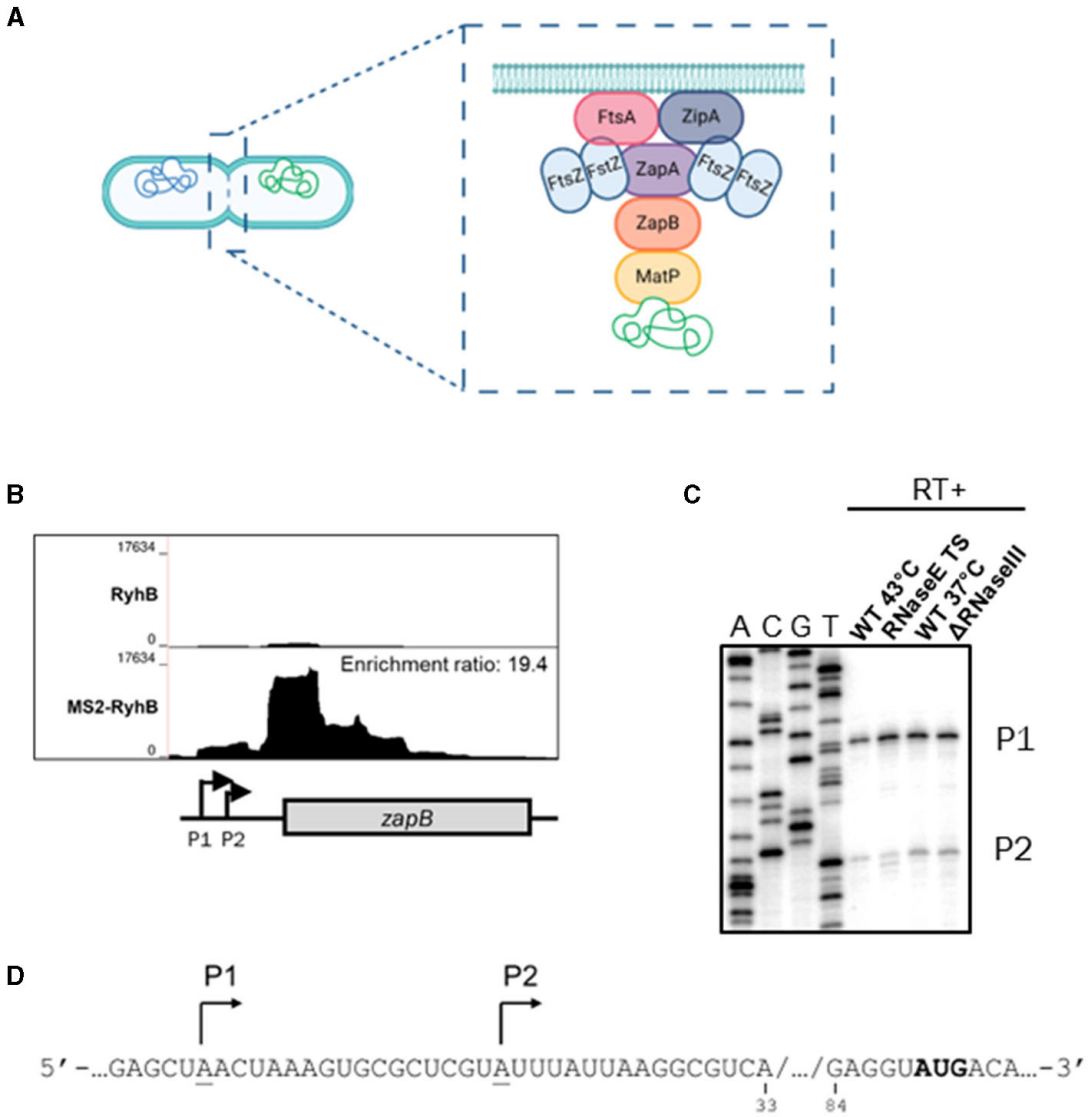
Identification and characterization of *zapB* mRNA as a putative target of RyhB. **(A)** Schematic view of cytokinesis with an emphasis on the divisome machinery. ZapB protein interacts with both ZapA (membrane) and MatP (chromosome). Created in BioRender. **(B)** Bedgraph of MS2-RyhB vs RyhB (control) samples from the MAPS technique, over the genomic region of *zapB*. Enrichment ratio of MS2-RyhB/RyhB = 19.4. **(C)** Primer extension assay of 5′-end radiolabeled *zapB* in different mutants of RNases. Total RNA was extracted from cells grown in LB medium at OD6_00nm_ = 1.0. A: sequencing ladder for adenine, C: sequencing ladder for cytosine, G: sequencing ladder for guanine, and T: sequencing ladder for thymine. P1: promoter 1 and P2: promoter 2. **(D)** Sequence of 5′UTR *zapB* mRNA. Validated promoters P1 and P2 of *zapB* are underlined and indicated with arrows.

The divisome complex is assembled around the FtsZ protein, which forms the Z-ring. The Z-ring formation starts with FtsZ polymerization, a tubulin homolog that recruits ZapAB and other divisome proteins to the septum (Adams and Errington, 2009; Galli and Gerdes, 2012). The Z-ring-associated protein ZapB plays a central role in the cell division process as it also coordinates chromosome segregation by interacting with MatP, a factor required for the terminus region of *E. coli* chromosome (Männik et al., 2016; see Figure 1A). MatP-ZapB interaction is necessary to anchor the Ter macrodomain at midcell (Espéli et al., 2012), thus positioning the Z-ring at the right place (Bailey et al., 2014). As expected for septation protein, mutant of *zapB* gene results in longer cells (Ebersbach et al., 2008). There are still many questions remaining concerning the molecular mechanisms of cell division, such as specific recruitment of divisome proteins in time and space. Moreover, different factors can influence bacterial cell division rates like nutrient availability (reviewed in Kellogg and Anne Levin, 2022).

Iron is an essential element for optimal growth and fitness of bacterial species. Iron is used in the electron transport chain to generate ATP and is central to the synthesis of amino acids and nucleotides. It is key to the transport of oxygen and contributes to cellular respiration (Messenger and Barclay, 1983; Andrews et al., 2003; Frawley and Fang, 2014). Iron also enables the expression of genes involved in iron uptake, storage, and utilization through the transcriptional regulator Fur (Hantke, 2001). Furthermore, iron is critical for DNA replication and repair by providing efficient synthesis of dNTPs through the action of ribonucleotide reductase. For instance, certain ribonucleotide reductases employ a variety of co-factors, ranging from di-iron cofactor (Fe-O-Fe) to Fe-S cluster, that catalyzes the conversion of ribonucleotide into deoxyribonucleotide (Puig et al., 2017). Moreover, DNA processing requires a series of Fe-containing protein to maintain genomic integrity. Many DNA-binding protein involved in base excision repair, endonuclease III, helicase, DNA primase, as well as DNA and RNA polymerases require Fe-S cluster for their activity (Puig et al., 2017; Grodick et al., 2014).

Previously, it was demonstrated that iron starvation, induced by various chelators, effectively blocked bacterial cell division (Santos et al., 2018). The observed phenotype indicated a significant increase (30–40%) in *E. coli* cell length. While the mechanism is not clear, formation of the cell division machinery was clearly affected in absence of iron. These results suggest a coordinated response between iron availability and the cytokinesis process. Nevertheless, iron starvation may create an unfavorable condition for the function of many iron-dependent enzymes, which prevents growth.

Absence of iron is one of the many stresses to which bacterial cells must adapt to thrive. To efficiently adjust their metabolism according to environmental insults, bacteria use small regulatory RNAs (sRNAs). These molecules extend usually between 100 and 500 nucleotides and can rapidly regulate gene expression to overcome different environmental stress. sRNAs that regulate gene expression at post-transcriptional level typically bind to their mRNA targets and either induce degradation of the mRNA by recruiting the RNA degradosome or activate their target mRNA translation by disrupting secondary structures and releasing the RBS (Hör et al., 2020; Lalaouna et al., 2013).

In *E. coli*, RyhB sRNA is expressed under iron-limiting conditions and mainly regulates iron-using proteins that are specifically involved in metabolism (*sodB* and *cysE*), TCA cycle (*acnB, sdhCDAB*, and *fumA*), iron metabolism (*iscRSUA* and *erpA*), and iron transport (*shiA* and *cirA*; Massé et al., 2005; Salvail et al., 2010; Salvail and Massé, 2012; Salvail et al., 2013; Chareyre and Mandin, 2018). Our group previously observed that cells lacking RyhB exhibited severe growth limitation during iron starvation (Jacques et al., 2006). However, the mechanism underlying this observation remains unclear.

In this study, our results suggest that the sRNA RyhB directly downregulates ZapB translation during iron starvation. Notably, ZapB is most likely the first target of RyhB not involved in iron metabolism, does not carry an iron co-factor, and the first involved in cytokinesis and DNA segregation. Reduction of ZapB expression by RyhB in wild-type cells correlates with longer cells, as compared to *ryhB* mutant, potentially due to reduced cell division. Our observations on the growth of Δ*ryhB* and Δ*ryhB*Δ*zapB* mutants suggest that absence of ZapB helps recover the reduced growth of Δ*ryhB* mutant during iron starvation. Furthermore, data from flow cytometry indicate that ZapB repression by RyhB resulted in increased number of chromosomes in cells growing under iron starvation. Additional observations suggest that helicase paralogs DinG and YoaA, both containing Fe-S clusters, might depend on RyhB activity for successful chromosome replication. Overall, our study suggests a possible mechanism of coordination between intracellular iron and cell division mediated by the sRNA RyhB, potentially by reducing cytokinesis when iron is limiting. This mechanism might also create safeguards for facilitating DNA replication and chromosome segregation by keeping Fe-S-dependent DNA helicases operational.

## Results

### Screening for new potential targets of RyhB

As previously described in Lalaouna et al. (2015) we used a tagged MS2-RyhB sRNA to co-purify and identify target mRNA partners. We re-examined the data from this experiment and found a new potential target: *zapB* mRNA. Figure 1B shows the enrichment of MS2-RyhB in the 5′ region of *zapB* mRNA. Moreover, RyhB and *zapB* interaction was also recovered in the RIL-seq analysis under iron limiting conditions and RNase E-CLASH (Melamed et al., 2016; Waters et al., 2017). We then characterized *zapB* transcription start sites by primer extension (Figure 1C and Supplementary Figure 1) and found 2 distinct promoters. When RNase E TS (thermosensitive) is inactivated using incubation at high temperature and RNase III mutated, the P1 and P2 are still clear. These results suggest that they are not cleavage sites. It is also interesting to observe that the −10 and −35 boxes of P1 have higher homology to the consensus sequence (Supplementary Figure 1B), which suggests that P1 is seemingly the primary transcription site (Figure 1D).

### RyhB regulates *zapB* mRNA at the translational level

**Figure 2:**
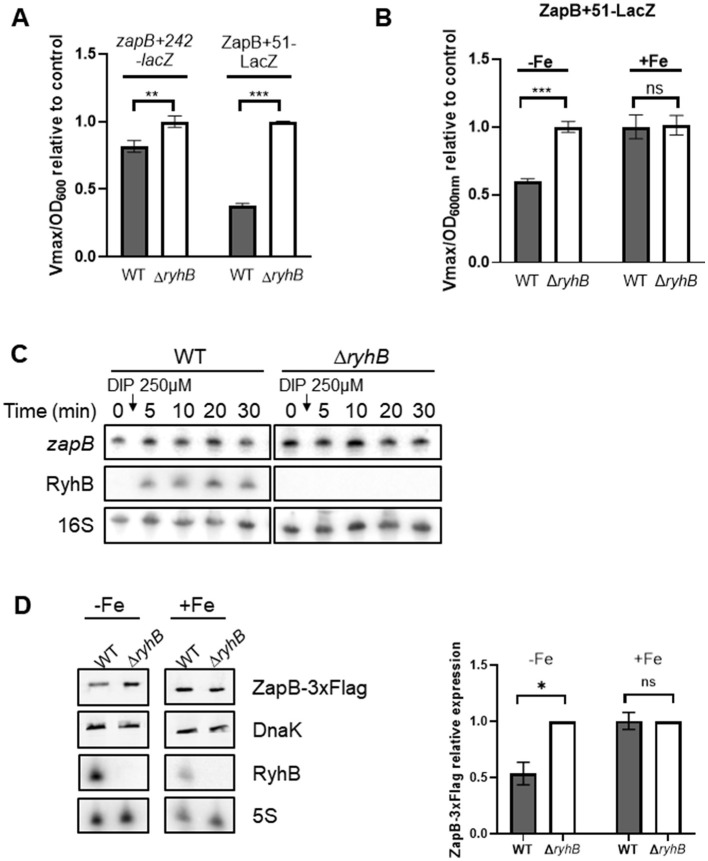
RyhB downregulates *zapB* mRNA, mostly through translational repression and does not induce degradation of this mRNA. **(A)** β-galactosidase assays using transcriptional *zapB*+*242-lacZ* or translational ZapB+51-LacZ fusions in LB media. RyhB expression was induced by addition of 250 μM 2,2′-dipyridyl at OD_600nm_ = 0.1 in WT or Δ*ryhB* background. Samples were taken at an OD_600nm_ = 1.5. Data represents two independent experiments. Student *t*-test with Welch's correction (**p = 0.0062 and ***p = 0.0003). **(B)** β-galactosidase assays using translational ZapB+51-LacZ fusion (WT or Δ*ryhB* background) in M63 minimal media, supplemented or not with 1 μM FeSO_4_ (Fe). Samples were taken at an OD_600nm_ = 0.2–0.35. Data represents three independent experiments. Student *t*-test with Welch's correction (***p = 0.0008). **(C)** Northern blot of *zapB* mRNA and RyhB following addition of 250 μM 2,2′-dipyridyl (DIP) at OD_600nm_ = 0.1 in WT or Δ*ryhB* background. 16S rRNA was used as a loading control. Data are representative of three independent experiments. **(D)** Western blot of ZapB-3xFlag in M63 minimal media (WT or Δ*ryhB* background), supplemented or not with 1 μM FeSO_4_ (Fe). Samples were taken at OD_600nm_ = 0.5. DnaK protein was used as a loading control. Northern blot of RyhB was performed at the same time and 5S rRNA was used as a loading control. Data represents two independent experiments. Student *t*-test with Welch's correction (*p = 0.0228).

We then addressed the regulation of expression exerted by RyhB on *zapB* mRNA. Gene reporter assays were performed using transcriptional *zapB*+*242-lacZ* and translational ZapB+51-LacZ. We used a transcriptional *zapB*+*242-lacZ* construct because of the presence of a cleavage site within the sequence, which would indicate RyhB-induced degradation. In contrast, the ZapB+51-LacZ has no cleavage site and thus indicate the effect of RyhB on *zapB* translation. These constructs show that RyhB downregulates *zapB* when cells are grown in the presence of 250 μM of the iron chelator 2,2′-dipyridyl (Figure 2A). Our results suggest that RyhB exerted about 60% repression on the translational fusion in presence of RyhB, in comparison to 20% repression on the transcriptional fusion. The same regulatory effects can be observed when overexpressing RyhB sRNA (Supplementary Figure 2A). We also compared the translational ZapB+51-LacZ fusion in WT and Δ*ryhB* cells grown in the presence and in the absence of iron. As shown in Figure 2B, WT cells display a strong repression in the absence of iron as compared to Δ*ryhB* cells. By using Northern blot, we also observed that RyhB does not induce rapid degradation of *zapB* mRNA (Figure 2C). This contrasts with previously characterized RyhB targets such as *sodB* or *sdhCDAB* mRNAs (Massé et al., 2003; Massé and Gottesman, 2002). However, we observed some degradation of *zapB* mRNA when inducing the sRNA RyhB for multiple hours (Supplementary Figures 2C, D). Since RyhB regulation seems to be mostly at the translational level, we constructed a ZapB-3xFlag strain to monitor ZapB proteins levels. By using Western blot, a significant decrease of ZapB-3xFlag protein can be observed in WT cells as compared to Δ*ryhB* cells (Figure 2D). Altogether, these results suggest that RyhB downregulates *zapB* mRNA, mostly at the translational level.

### RyhB binds at two distinct sites within *zapB* mRNA

**Figure 3:**
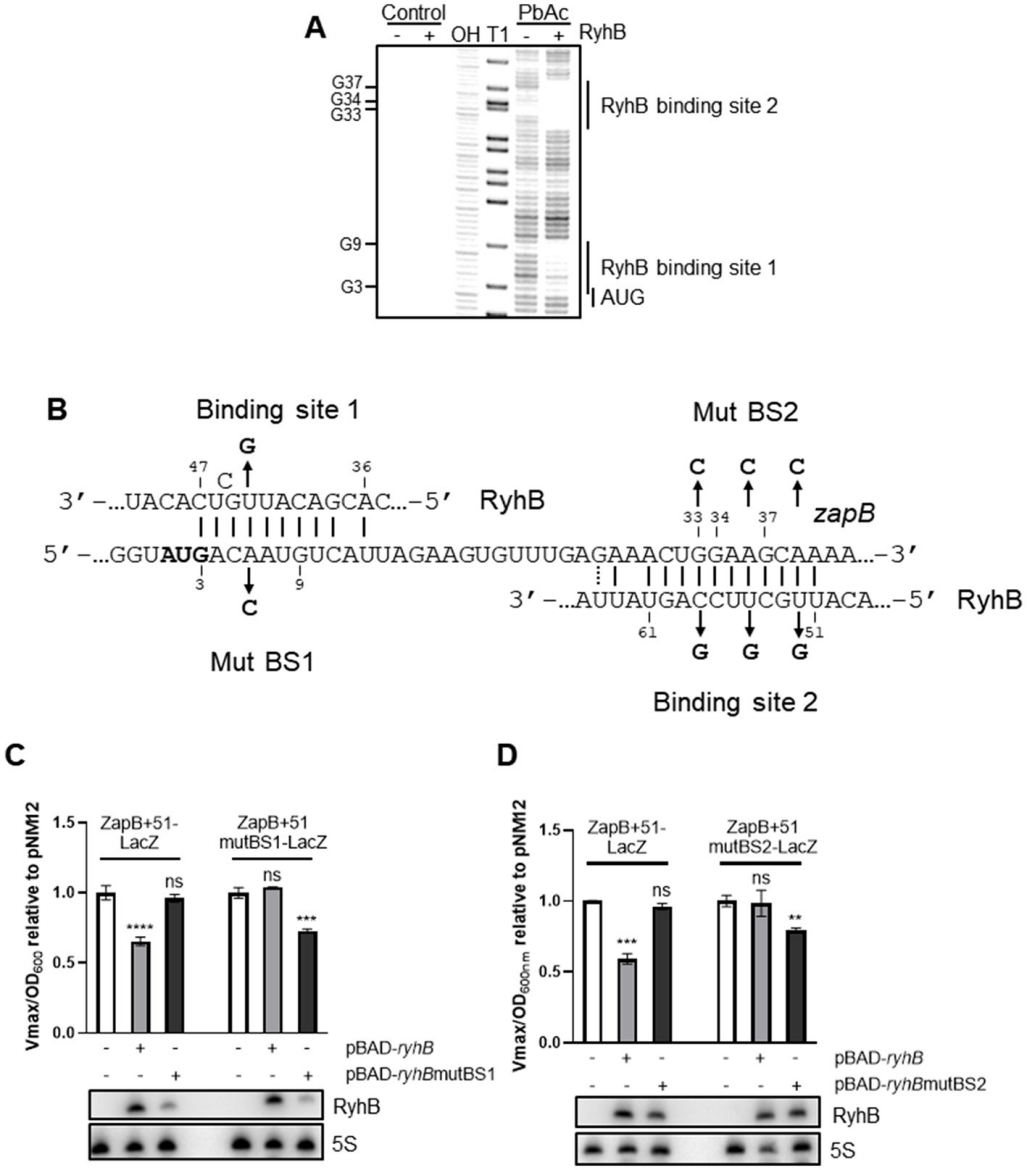
RyhB directly base pairs in the coding sequence of *zapB* and both binding sites independently regulate *zapB*. **(A)** Lead acetate (PbAc) probing assay of 5′-end radiolabelled *zapB*, in the presence or absence of RyhB. OH: alkaline ladder, T1: RNase T1 ladder. **(B)** Validated pairing between RyhB sRNA and *zapB* mRNA mapped with *in vitro* probing. The numbers are in reference to the *zapB* promoter P1 being 1. The start codon of *zapB* is in bold and mutations in RyhB and *zapB* are indicated. **(C)** β-galactosidase assays using the ZapB+51-LacZ and ZapB+51mutBS1-LacZ **(C)** or ZapB+51mutBS2-LacZ **(D)** translational fusions in Δ*ryhB* background. Expression of RyhB, RyhBmutBS1, or RyhBmutBS2 from pBAD promoter was induced by addition of 0.1% arabinose at OD_600nm_ = 0.1. Samples were taken at OD_600nm_ = 1.5. pNM12 plasmid was used as a control. Northern blot assays were performed at the same time to monitor levels of RyhB sRNA. 5S rRNA was used as a loading control. Data represents three independent experiments. Two-way ANOVA with Dunnett's multiple comparisons test using pNM12 as control (**p = 0.0078, ***p = 0.0002, and ****p < 0.0001).

To delineate the exact position where RyhB interacts with the *zapB* transcript, we performed a lead acetate probing assay. Remarkably, Figures 3A, B show that RyhB binds at two distinct sites on *zapB* mRNA. The first binding site is immediately downstream of the initiating AUG and the second binding site starts at the 9th codon within the coding sequence (CDS). Interestingly, as Figure 3B shows, *zapB* binding sites interact with distinct regions of RyhB sRNA.

To investigate whether the two RyhB binding sites on *zapB* mRNA are responsible for regulation of translation, point mutations were introduced on RyhB, which was overexpressed from a plasmid with an arabinose-inducible promoter (Figure 3B). The compensatory point mutations were also introduced on the translational ZapB+51-LacZ fusions and β-galactosidase assays were performed. As shown in Figure 3C, when RyhB is mutated at the binding site 1 (BS1), no regulation on *zapB* is observed. However, when the complementary mutant is introduced on *zapB*, we fully recover the repression of RyhB on *zapB* mRNA. We confirmed by Northern blot that RyhB and its mutated version were normally expressed under those conditions (Figures 3C, D). The same experiments were performed with RyhB binding site 2 (BS2) on *zapB*. The 2nd binding site exerts a weaker repression of ZapB at translational level (about 25% repression). We also performed the experiments with mutations of both binding sites on RyhB (Supplementary Figure 3) and show that the regulatory effect of each binding site is dependent from each other.

### RyhB-*zapB* interaction impacts cell length

**Figure 4:**
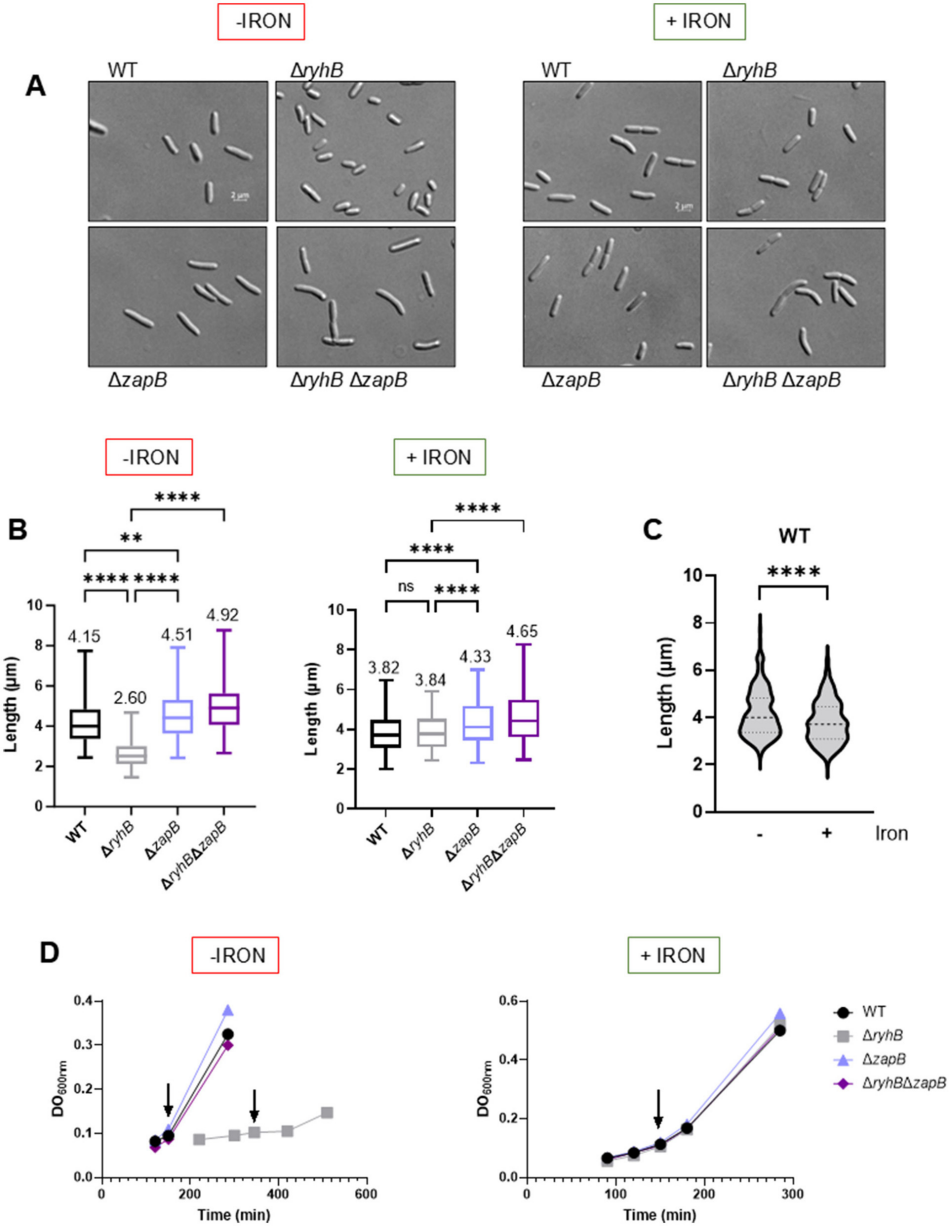
RyhB affects cell length in *E. coli* cells by regulating *zapB* mRNA. *E. coli* K12 MG1655 WT, Δ*ryhB*, Δ*zapB*, and Δ*ryhB* Δ*zapB* cells grown in chelex-treated M63 minimal media supplemented or not with 1 μM FeSO_4_ (+/- iron). **(A)** Phase-contrast microscopy images. Samples were collected at OD_600nm_ = 0.1 and images were taken with Zeiss Axio Observer Z1 microscope at 1000X. **(B)** Cell lengths count from 300 cells resulting from images of **(A)** with Zen 3.3 software. The mean of cell lengths is indicated above each strain. Two-way ANOVA with Tukey's multiple comparisons test (****p < 0.0001 and **p = 0.0012). **(C)** Representation of the WT strain with or without 1 μM FeSO4 in chelex-treated M63 minimal media from **(B)**. Student *t*-test with Welch's correction (****p < 0.0001). **(D)** Growth curves of the different strains. Arrows indicated when samples were taken from microscopy images.

Because ZapB protein is involved in chromosome segregation and cytokinesis processes, we investigated how RyhB regulation on *zapB* mRNA could impact these cellular functions. First, we performed microscopy experiments to assess cell length in different genetic backgrounds. We expected that under iron starvation, WT cells could be longer since RyhB is expressed and downregulates *zapB*, which leads to reduced cell division (Ebersbach et al., 2008). In fact, Figures 4A, B show that in absence of iron, the WT strain exhibits cells that are 40% longer than those from the *ryhB* mutant strain. Strikingly, deleting the *zapB* gene helps Δ*ryhB* cells to recover to a slightly longer length than WT. We also show that a deletion of *zapB* alone leads to longer cells as previously demonstrated by another group (Ebersbach et al., 2008). When adding 1 μM of FeSO_4_ in the culture media (Figures 4A, B, right panels), we completely lose the reduced length phenotype of the Δ*ryhB* cells. We also observed on Figure 4C that a WT strain presents longer cells in absence of iron, suggesting that this phenotype is linked to iron availability and RyhB expression. Interestingly, we observed that the Δ*ryhB*Δ*zapB* null strain grows like a WT and does not have the growth phenotype that the Δ*ryhB* strain harbors under iron starvation (Figure 4D, left panel), as previously described by our group (Jacques et al., 2006). These results indicate that absence of *zapB* reduces both phenotypes of short length and slow growth of the *ryhB* mutant when cells are grown in absence of iron.

### Chromosome segregation is also affected by RyhB-*zapB* interaction

**Figure 5:**
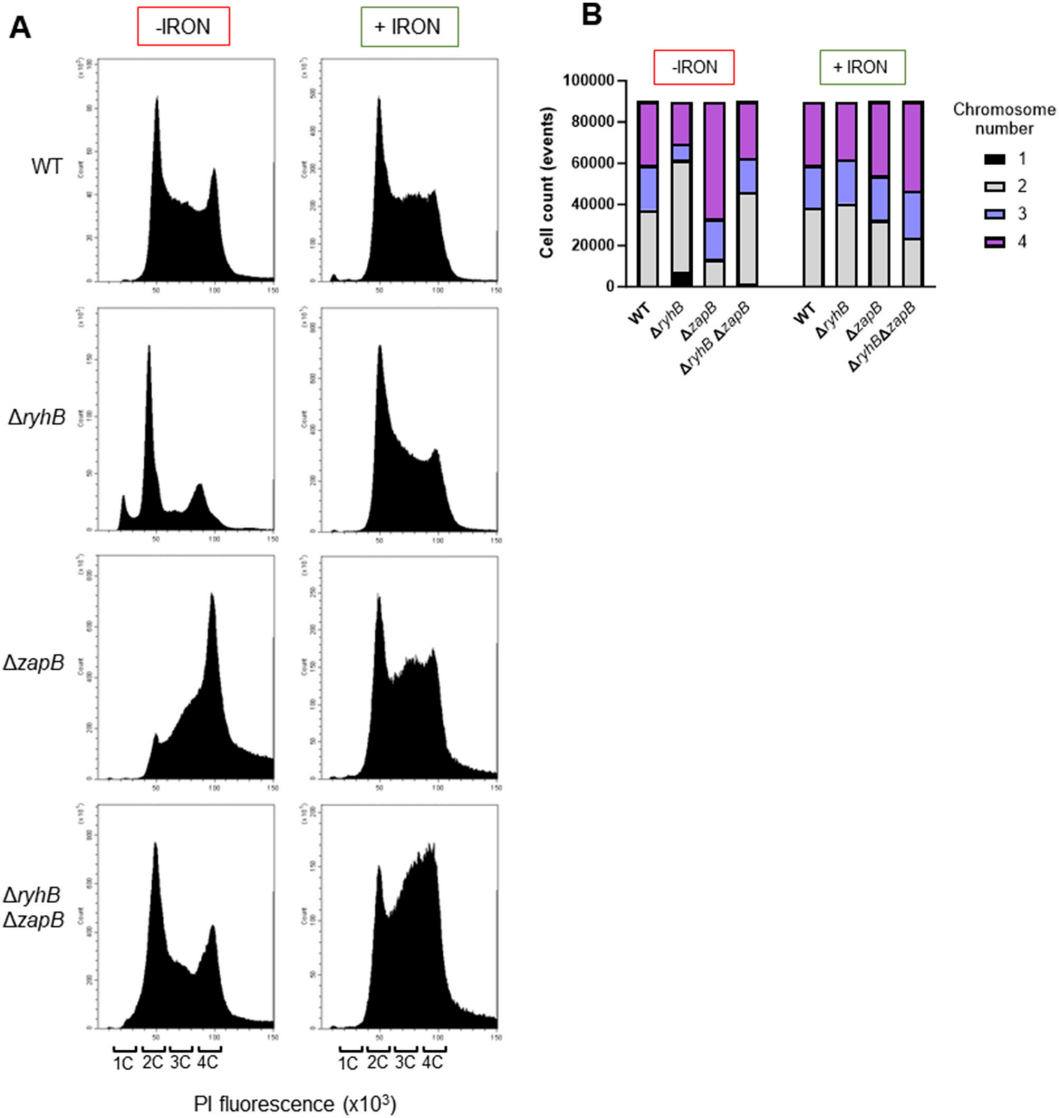
RyhB also affects chromosome segregation in *E. coli* by regulating *zapB* mRNA. **(A)** Flow cytometry assay in chelex-treated M63 minimal media, supplemented or not with 1 μM FeSO_4_ (+/- iron). Graphics show DNA distribution in WT, Δ*ryhB*, Δ*zapB*, and Δ*ryhB* Δ*zapB* cells at OD_600nm_ = 0.1 and stained with propidium iodide (PI). 1C: 1 chromosome in a cell, 2C: 2 chromosomes in a cell, 3C: 3 chromosomes in a cell and 4C: 4 chromosomes in a cell. **(B)** DNA distribution from **(A)** normalized to 90,000 cells for each background (WT, Δ*ryhB*, Δ*zapB*, and Δ*ryhB* Δ*zapB*).

Because ZapB protein is also involved in chromosome segregation, we performed flow cytometry experiments to assess chromosome distribution in strains previously used for microscopy experiments above. We observed that the Δ*ryhB* strain exhibits a lower chromosome number (1 or 2) than the other strains (WT, Δ*zapB*, and Δ*ryhB*Δ*zapB*) when grown under iron starvation (Figures 5A, B). This result coincides with the observation that a Δ*ryhB* strain presents shorter cell in this condition as shown in our microscopy experiments. We also observed that a Δ*zapB* strain, grown in absence of iron, has more cells containing 4 chromosomes compared to other backgrounds, which correlates with the longer cell length phenotype previously observed. Interestingly, in absence of iron, a WT strain presents a peak representing 4 chromosomes (Figure 5A) which is absent when adding iron to the media. Moreover, the Δ*ryhB*Δ*zapB* strain also presents a similar chromosome distribution to a WT strain. However, when adding 1 μM FeSO_4_ to the media, the chromosome profile of each background becomes similar, which suggests that the chromosome distribution phenotype depends on iron availability. Altogether, these results demonstrate that deleting the *zapB* gene helps recover the chromosome distribution of the Δ*ryhB* strain.

### DNA replication of Δ*ryhB* strain is affected by azidothymidine and rifampicin treatments

**Figure 6:**
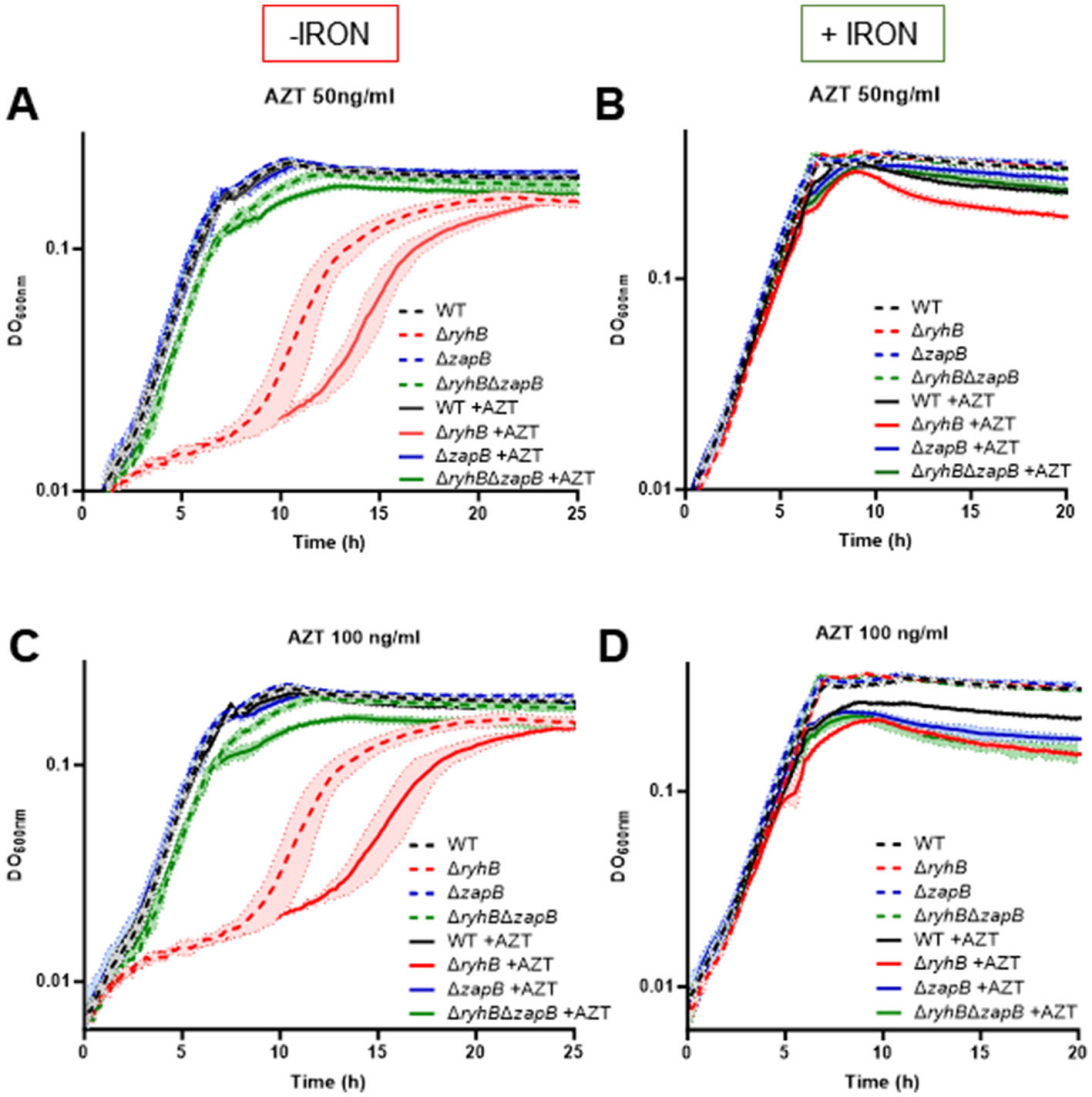
Absence of RyhB and iron starvation affects DNA replication machinery, which leads to cell growth inhibition. Growth curves in M63 minimal media supplemented or not with 1 μM FeSO_4_ (+/- iron). Azidothymidine (AZT) was added at 50 ng/ml **(A, B)** or 100 ng/ml **(C, D)**. WT: black, Δ*ryhB*: red, Δ*zapB*: blue and Δ*ryhB* Δ*zapB*: green. Dotted lines represent absence of AZT and full lines represent presence of AZT. Data represent three replicates with standard deviation.

Next, we wanted to assess whether a Δ*ryhB* strain is affected by azidothymidine (AZT). AZT is an analog of thymidine which blocks the elongation of the DNA replication machinery. AZT was already shown to be involved with YoaA and DinG activities (Brown et al., 2015), two iron-using DNA helicases and paralogs essential for the function of the DNA replication machinery. The absence of YoaA or DinG exacerbated the sensitivity to AZT. As shown in Figures 6A, C, the Δ*ryhB* strain is clearly affected by the presence of AZT in absence of iron. Remarkably, this strain seems to be the only one strongly affected by the presence of AZT. Notably, when adding iron and AZT (Figure 6B), the deleterious effect of AZT on Δ*ryhB* strain is clearly lost. We also observed that all strains become more sensitive to AZT at high concentration during stationary phase in the presence of iron (Figure 6D). This correlates with previous observations from our lab suggesting there is less intracellular iron when cells enter the stationary phase of growth (Jacques et al., 2006).

Because of the DNA replication problem suggested by our AZT assay above, we also monitored the mutation rate of rifampicin resistance in Δ*ryhB* strain. As seen in Supplementary Figure 6, the Δ*ryhB* strain presents higher mutation rate following rifampicin treatment as compared to WT strain. Rifampicin targets the β-subunit of the RNA polymerase by inducing mutations in RNA polymerase (Wehrli, 1983), which suggests that the fidelity of DNA replication is also affected when RyhB is absent. The Δ*ryhB*Δ*zapB* strain also recovers this phenotype and contains about the same mutation rate than a WT strain. Altogether, these results suggest that DNA replication machinery is affected in the Δ*ryhB* strain along with the chromosome distribution and cell length.

## Discussion

**Figure 7:**
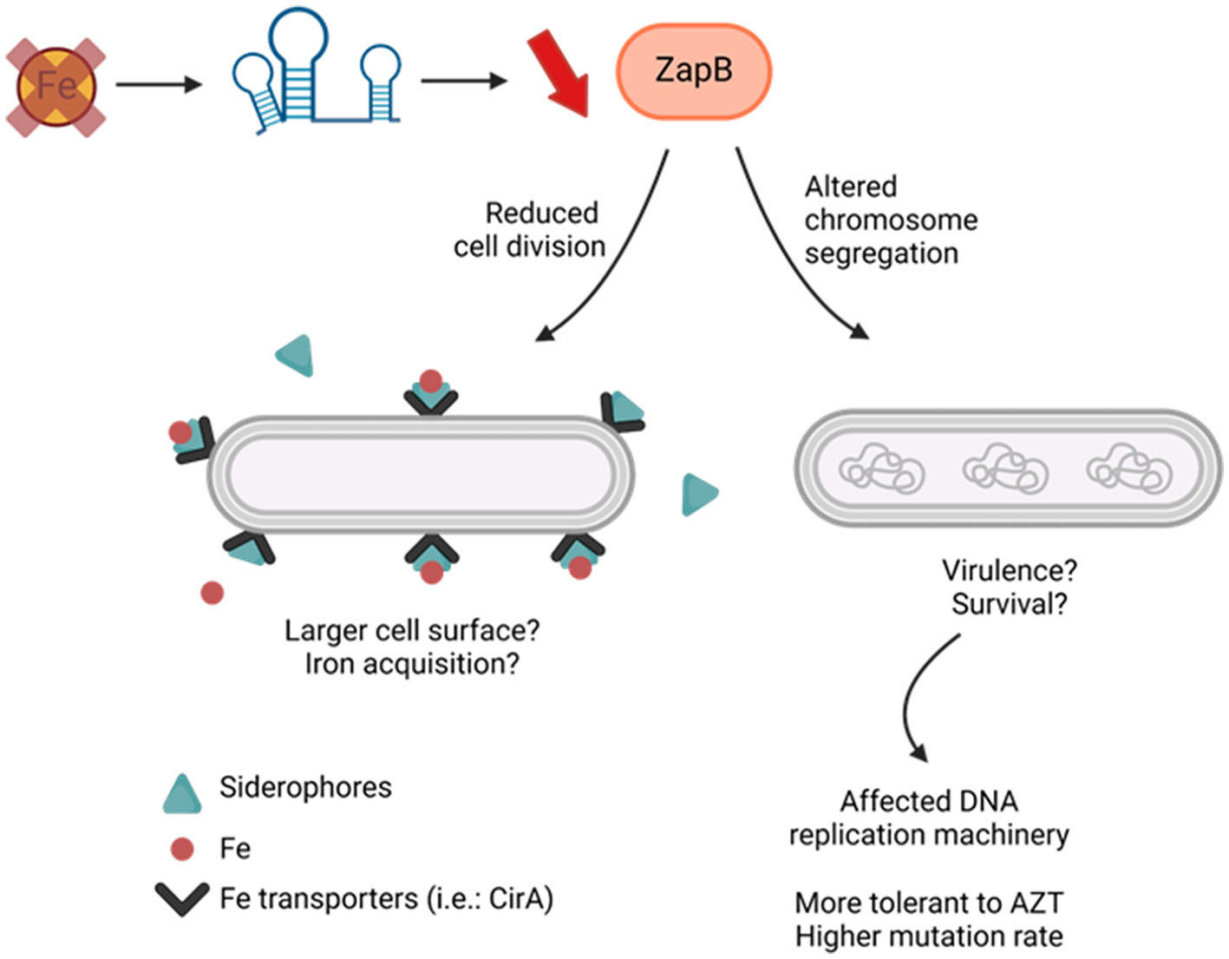
Proposed model for RyhB-*zapB* interaction impacts on *E. coli* cells. Iron starvation in bacterial environment induces RyhB expression which inhibits *zapB* mRNA, mostly at translational level. Lower levels of ZapB protein leads to reduced cell division and altered chromosome segregation in *E. coli* cells. This results in longer cells with affected DNA replication machinery. Created in BioRender.

Most organisms growing under iron starvation require extensive metabolic adaptation to palliate and optimize growth in the absence of this key element. Previous studies indicated that the Krebs cycle, respiratory chain complex, and metabolite trans-membrane transport are particularly affected during iron starvation (reviewed in Chareyre and Mandin, 2018). A more recent study added cell division as another cell function impacted by absence of iron (Santos et al., 2018). Our observations corroborate with the description that iron starvation decreases *E. coli* cytokinesis and suggest a mechanism in which the sRNA RyhB reduces the expression of ZapB. Remarkably, repression of the factor ZapB through RyhB-*zapB* interaction not only impacts cell length but also chromosome distribution within a bacterial population. Moreover, our data showed that RyhB-deficient cells growing in absence of iron exhibit increased sensitivity to AZT, a drug targeting Fe-S enzymes involved in the DNA replication machinery. Interestingly, deletion of the *zapB* gene leads Δ*ryhB* strain to recover normal growth in iron depleted conditions. What's more, the Δ*ryhB*Δ*zapB* strain shows similar recovery of both chromosome distribution and AZT resistance as compared to Δ*ryhB* strain. Altogether, these results suggest a possible link between cytokinesis, chromosome segregation, and iron availability based on RyhB-*zapB* interaction (Figure 7).

Santos et al. (2018) previously demonstrated that both assembly and localization of the cell divisome, at the late stages of cytokinesis, were affected during iron starvation. Moreover, iron chelation caused a reduction of fluorescent signals in ZapA, FtsN, FtsK, and FtsI proteins tagged with GFP, suggesting a reduced expression in parts of the divisome machinery. Although the authors did not specifically investigate ZapB protein, it had already been shown that ZapB directly interacts with ZapA (Galli, 2011; Buss et al., 2017). Our own results suggest that, under restricted iron levels, the sRNA RyhB reduces the expression of ZapB, thereby reducing cell division and affecting the functional divisome machinery. Our present work supports the idea that observations made by Santos et al. were due to ZapB repression by RyhB in the absence of iron.

Our laboratory has previously shown that a Δ*ryhB* strain had a deficient growth in minimal media without iron (Jacques et al., 2006). While this suggests that RyhB is essential for optimal growth under iron restricted condition, it remained unclear why cells struggled in absence of the sRNA. We assumed that absence of regulation of several target mRNAs by RyhB could result in growth struggle. Our new data suggests that this growth phenotype could be the consequence of a single target, *zapB*, being misregulated in absence of iron, which results in premature cell division, giving rise to shorter cells and abnormal chromosome segregation.

The Δ*ryhB* growth defect under restricted iron could also be resulting from fewer chromosomes and DNA deficiencies. Data from flow cytometry (Figure 4B) indicate that the Δ*ryhB* mutant exhibits the most cells with only one or two chromosomes while having the least cells with 3 or 4 chromosomes as compared to WT, Δ*zapB*, and Δ*ryhB*Δ*zapB* backgrounds. Additional observations we made on the Δ*zapB* strain in Figures 4, 5 also correlate with previous observations made by Ebersbach et al. (2008), which stated that a deletion of *zapB* increases the average cell length. Our results suggest that a Δ*zapB* mutant presents longer cells and additional chromosomes per cell, which can be observed in Figure 5A by a shift in the chromosome profiles in absence of iron. Moreover, our control experiment in the presence of rifampicin and cephalexin to stop DNA replication (through RNA polymerase inhibition) and cell division supports this observation (Supplementary Figure 5).

Notably, WT cells growing in the absence of iron display an increase in chromosome number as compared with cells grown in the presence of iron (Figure 5A, WT +/- iron). This increase in chromosome number in absence of iron probably stems from RyhB repressing ZapB expression as the *zapB* mutant also shows increase in chromosome number (Figure 5A, Δ*zapB* strain, -iron). Previous reports described how the chromosome number usually follows a trend correlating with growth rate (reviewed in Reyes-Lamothe et al., 2012; Sanders et al., 2023). This higher number of chromosomes would allegedly prepare rapidly growing cells to divide at higher rate. In our observations however, the higher number of cells with four chromosomes correlates with a situation where iron is scarce and growth rate is low. This situation is similar to UPEC infecting the host bladder cells where significantly longer bacterial cells were observed (Justice et al., 2004, 2006). Surprisingly, septation of these filamented cells on the bladder surface favored infection. Moreover, a recent study reported that overproducing the iron-using IscA protein leads *E. coli* cells to filament (Wei et al., 2024). These observations can be explained by sequestration of free intracellular iron when overproducing the protein IscA, thus effectively inducing cellular iron starvation and RyhB expression. Notably, the *zapA* transcript, encoding a factor interacting with ZapB, was strongly induced during these conditions, which indicate that other proteins of the divisome can potentially affect cytokinesis during iron starvation. Overproduction of ZapA was previously shown to generate filamentation of *E. coli* cells (Galli and Gerdes, 2012).

Repression of another septation protein has been shown in previous work, where the sRNA DicF can repress translation of the *ftsZ* transcript (Tétart et al., 1992; Balasubramanian et al., 2016). Although the physiological reason for this regulation remains unclear, it was shown that anaerobic conditions increased DicF stability and permitted the repression of *ftsZ* translation (Murashko and Lin-Chao, 2017). These results indicate how the bacterial cell can change its shape to adapt to environmental challenges similar to our results in absence of iron.

To our knowledge, YoaA, DinG, and QueE are the only proteins involved in cell division or chromosome segregation which are using iron. Notably in *E. coli*, paralogs YoaA, and DinG encode for DNA helicases, both of which use 4Fe-4S clusters as prosthetic groups (Bak and Weerapana, 2023; Ren et al., 2009). We previously demonstrated that a Δ*ryhB* mutant contains less intracellular iron than WT cells growing under iron starvation conditions (Salvail et al., 2010). From this perspective it is tempting to hypothesize that, under these conditions (iron restriction or absence of RyhB), intracellular iron becomes too limited to sustain YoaA and DinG enzymatic activity. This reduced YoaA and DinG enzymatic activity in absence of iron can potentially lead to sensitivity to azidothymidine drug (AZT), an analog of thymidine which blocks DNA replication, as previously demonstrated in Δ*yoaA* and Δ*dinG* strains (Brown et al., 2015). Our results demonstrated that a Δ*ryhB* strain growing in absence of iron is more sensitive to AZT. This suggests that in conditions of iron starvation, and potentially reduced YoaA and DinG activities, cells might benefit from reduced division through repression of ZapB protein by RyhB. Even though *zapB* is potentially the first target of RyhB not directly involved in iron metabolism, reducing its expression might create an environment facilitating DNA replication and chromosome segregation. Another reason why RyhB could downregulate ZapB in an iron starved environment would be that longer cells expose more surface for iron acquisition machinery and siderophores transport.

Finally, this regulation of RyhB on *zapB* mRNA seems peculiar as RyhB usually rapidly degrades its targets within minutes of iron depletion induction (Massé et al., 2003; Massé and Gottesman, 2002). It is interesting to note that in the RIL-seq experiment (Melamed et al., 2016), the interaction between RyhB and *zapB* depends on Hfq pulldown, suggesting that Hfq is involved in this interaction. In the case of RyhB-*zapB* regulation, it is mostly at translational level and the mRNA level remains stable throughout a 30-min induction of iron depletion. This could be explained by the fact that RyhB has two binding sites on *zapB* mRNA, similar to RprA regulation of *hdeD* mRNA (Lalaouna et al., 2018). We also hypothesize that two RyhB molecules are needed to bind *zapB* mRNA due to the opposite 5′-3′ sense of its binding regions. The first binding site explains the translation block since it's located near the translation start codon of ZapB. The second binding site, located in *zapB* CDS, could block a nuclease site which could explain the regulatory effect on *zapB* transcription and *zapB* mRNA stability when RyhB is expressed. This hypothesis is also supported with the RNase E-CLASH experiment which indicated an interaction of RyhB::*zapB* along with RNase E at the second binding site (Waters et al., 2017). Notably, this suggests a functional regulation of RyhB on *zapB* transcript beyond the five-codon window already described by Bouvier et al. (2008). Altogether, our results demonstrate that RyhB not only regulates iron metabolism but DNA replication, chromosome segregation, and cell division as well.

## Methods

### Strains construction

#### Transcriptional and translational lacZ fusions

To generate transcriptional *zapB*+*242-lacZ* fusion and translational ZapB+51-LacZ fusion, PCR was performed with oligonucleotides EM2988-2989 and EM2988-3031, respectively, then digested with EcoRI and BamHI. Digested fragments were then ligated into EcoRI-BamHI digested pFRΔ or pRS1551 for transcriptional and translational fusion, respectively.

To generate translational ZapB+51-LacZmutBS1, ZapB+51-LacZmutBS2, and ZapB+51-LacZmutBS1+2 fusions, two independent PCR reactions were done with the following oligonucleotides: ZapB+51-LacZmutBS1 with EM194-4491 and EM4490-195, ZapB+51-LacZmutBS2 with EM194-4596 and EM4595-195, and ZapB+51-LacZmutBS1+2 with EM194-4596 and EM4595-EM195 using ZapB+51-LacZmutBS1 as template for PCR. The PCR fragments were digested with EcoRI and BamHI, then ligated into EcoRI-BamHI digested pRS1551. Numbers in the constructs represent nucleotides from start codon.

The *lacZ* fusions were then inserted as single copy into the chromosome of a Δ*ara714* strain (see Supplementary Table 1) at the λ attI site, as described previously (Simons et al., 1987). Lysogens were screened by PCR for selection of single insertion recombinant λ (Powell et al., 1994). The *lacZ* fusions were then transferred to a Δ*ryhB*::cat strain using P1 transduction. Plasmids (pNM12, pBAD-*ryhB*, pBAD-*ryhB*mutBS1, pBAD-*ryhB*mutBS2 and pBAD-*ryhB*mutBS1+2) were then inserted in the *lacZ* fusions strains using TSS transformation.

#### Plasmids

To generate pBAD-*ryhB*mutBS1, pBAD-*ryhB*mutBS2, and pBAD-*ryhB*mutBS1+2, two independent PCR reactions were performed with the following oligonucleotides: *ryhB*mutBS1 with EM168-EM4594 and EM4593-455, *ryhB*mutBS2 with EM168-4598 and EM4597-EM455, and *ryhB*mutBS1+2 with EM168-4743 and EM4742-EM455. A third PCR was then generated using the two PCR products as template with EM168-EM455 for each construct. The final PCR products and pNM12 plasmid were digested with EcoRI and MscI then ligated and inserted into strains DL1347, MG101, MG131, and MG141 (see Supplementary Table 1).

#### *zapB* chromosomal deletion

The chromosomal mutation of *zapB* was generated using the method described by Datsenko and Wanner (2000). The PCR reaction was done with EM3042-3043 on pKD4 to introduce a kanamycin resistance cassette. The PCR product was then transformed into DY330 after induction of λ Red. The construct was verified by sequencing and transferred to a Δ*ryhB*::cat strain by P1 transduction.

#### ZapB-3xFlag

The ZapB-3xFlag construct was obtained by transferring the 3x-Flag sequence to the chromosomal zapB gene using the method described by Datsenko and Wanner (2000). First, a PCR reaction was done with EM1689-EM3467 on pKD4 to introduce a kanamycin resistance cassette with 3xFlag sequence. This PCR product was used as a template for a second PCR reaction with EM3468-3467 to introduce *zapB* gene fused to the 3xFlag sequence. The PCR product was then transformed into DY330 after induction of λ Red. The construct was verified by sequencing and transferred to a Δ*ryhB*::cat strain by P1 transduction.

### Primer extension assay

Strains were diluted 1/1,000 in LB medium and grown in rotary shaker at 37°C. RNase E TS is thermosensitive and was inactivated at 43°C for 15 min. RNA samples were extracted using the classic hot-phenol chloroform protocol as described previously (Aiba et al., 1981) after cells reached OD 1.0. Primer extension was then performed following the protocol previously described (Beroual et al., 2022). 20 μg of total RNA was used with radiolabeled primer EM4953 to generate cDNA that was migrated on 8% acrylamide gel containing 8 M urea. The sequencing ladder was generated by PCR with radiolabeled probe EM4953 from DNA matrix using primers EM2988 and EM3045. Gel was exposed on phosphor screen and revealed with GE Healthcare Typhoon Trio.

### Lead acetate probing assay

Transcription was done with T7 polymerase from a DNA product generated by PCR with EM3281 and EM3282 for *zapB* and EM88 and EM89 for *ryhB*. 50 pmols of *zapB* transcript was radiolabeled and incubated in presence or not of 1 μM RyhB following the protocol described in Desnoyers et al. (2009). Samples were loaded on an 8% acrylamide gel containing 8 M urea, then exposed to phosphor screens and revealed with GE Healthcare Typhoon Trio.

### β-galactosidase assays

Strains were diluted 1/1,000 in LB medium or adjusted to OD_600nm_ = 0.04 in M63 minimal media supplemented with 0.2% glucose, in absence or presence of 1 μM FeSO_4_ and grown in rotary shaker at 37°C. RyhB sRNA expression was induced by addition of 250 μM a,a'-Dipyridyl (DIP from Fisher Scientific) or 0.1% arabinose (Ara). Then, β-galactosidase kinetic assays were performed as previously described (Majdalani et al., 1998) using SpectraMax 250 microtiter plate reader (Molecular Devices).

### RNA extraction and northern blot analysis

RNA samples were extracted following the classic hot-phenol protocol as described previously (Aiba et al., 1981). For northern blot analysis, 5–10 μg of total RNA were loaded on a 5–10% acrylamide gel containing 8 M urea. Electrotransfer was done on Hybond-XL membranes for 1 h at 200 mA, then crosslinked under 254 nm UV for 45 s. Pre-hybridization was done in Church buffer for 1 h at 42°C and radiolabeled DNA probes detailed in Supplementary Table 2 were added overnight. Membranes were then exposed to phosphor screens and revealed with GE Healthcare Typhoon Trio.

### Protein extraction and western blot analysis

Strains were adjusted to OD_600nm_ = 0.04 in M63 minimal media supplemented with 0.2% glucose, in absence or presence of 1 μM FeSO_4_ and grown in rotary shaker at 37°C. At OD_600nm_ = 0.5, proteins were extracted with 50% trichloroacetic acid. For western blot analysis, 10 μl of protein samples were loaded on 15% acrylamide gel. Electrotransfer was done on nitrocellulose membranes (Hybond-XL from GE Healthcare) for 1 h at 250 mA. Antibody anti-flag mouse (Sigma) diluted 1:1000 or antibody anti-DnaK mouse (Abcam) diluted 1:10 000 was added overnight and secondary antibody IRDye 800CW goat anti-mouse (LI-COR) was added for 1 h. Membranes were revealed with LI-COR and analyzed with Odyssey software.

### Microscopy

Cultures were adjusted to OD_600nm_ = 0.04 in chelex M63 minimal media supplemented with 0.2% glucose, in absence or presence of 1 μM FeSO_4_, and grown in rotary shaker at 37°C. When cells reached OD_600nm_ of 0.1, 1.5 ml of culture was centrifuged and resuspended in 50 μl PBS 1X. 5 μl of cells were placed on 2% agarose pad. Cells were imaged with Zeiss Axio Observer Z1 microscope, using phase-contrast settings at 1000X magnification and analyzed using Zen 3.3 software.

### Flow cytometry assay

Cultures were adjusted to OD_600nm_ = 0.04 in chelex M63 minimal media supplemented with 0.2% glucose, in absence or presence of 1 μM FeSO_4_, and grown in rotary shaker at 37°C. When cells reached OD_600nm_ of 0.1, cells were fixed with cold ethanol 70%, then stained with 0.1% Triton-X, 10 μg/ml RNaseA and 10 μg/ml propidium iodine on a rotor agitator. For the controls, cells were treated with 15 μg/ml cephalexin and 100 μg/ml rifampicin for 2 h at 37°C before cold ethanol fixation. Control cells were stained with LIVE/DEAD^TM^ Fixable Green Dead Cell Stain (Invitrogen, L34969) and added in each cell samples. Samples were run on CytoFLEX 20 (Beckman Coulter) and analyzed using CytExpert software.

### Growth curves

Overnight cultures were grown in M63 minimal media supplemented with 0.2% glucose in a rotary shaker at 37°C. Cultures were then adjusted to 6 x 10^7^ cells/ml in M63 minimal media supplemented with 0.2%glucose, in presence or absence of 1 μM FeSO_4_. 3′-Azido-3′-deoxythymidine (AZT, Sigma) was added to the cultures to the respective concentrations. The cultures were incubated at 37°C and OD_600nm_ was measured every 10 min using BioTek EPOCH2T microplate reader.

### Rifampicin plate assays

Cultures were adjusted to OD_600nm_ = 0.04 in M63 minimal media supplemented with 0.2% glucose and grown in rotary shaker at 37°C. When cells reached OD_600nm_ of 0.3, 10 ml of culture was centrifuged at 2,500 *g* for 15 min. The pellets were washed with 1 ml PBS 1X, then resuspended in 200 μl PBS 1X for plating on LB agar containing 100 μg/ml rifampicin. After overnight incubation at 37°C, colonies were counted and reported as mutation rate (CFU on LB-rifampicin/total CFU). Total CFUs were done by serial dilution in 0.9% NaCl and plating on LB agar.

## Data Availability

The original contributions presented in the study are included in the article/Supplementary material, further inquiries can be directed to the corresponding author.
