# Supplementary material for: Coordination of cell division and chromosome segregation by iron and a sRNA in Escherichia coli
Source: Front Microbiol. 2024 Oct 25;15:1493811. doi: 10.3389/fmicb.2024.1493811 (PMC11584013; doi:10.3389/fmicb.2024.1493811)
Supplement: Supplementary file 1 [file Data_Sheet_1.PDF]

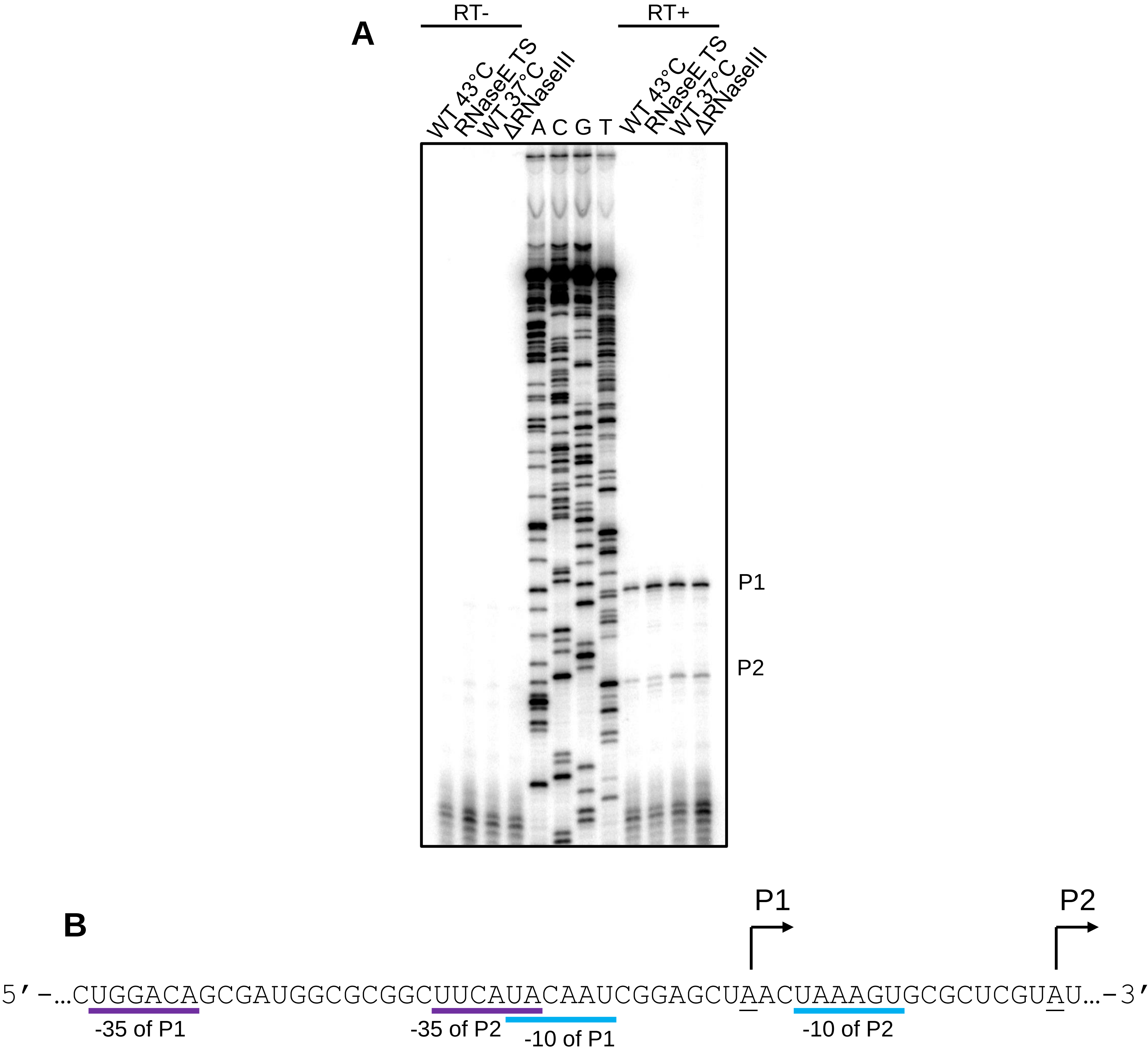

Figure S1. Identification of *zapB* gene promoters. A) Primer extension assay of radiolabelled *zapB* mRNA extracted from different mutants grown in LB media at OD_{600nm}=1.0. Controls RT- lanes do not contain reverse transcriptase. A: sequencing ladder for adenine, C: sequencing ladder for cytosine, G: sequencing ladder for guanine and T: sequencing ladder for thymine. P1: promoter 1 and P2: promoter 2. **B)** Sequence of 5’UTR *zapB* mRNA. Validated promoters P1 and P2 of *zapB* are underlined and indicated with arrows. -10 and -35 boxes are underlined in blue and purple, respectively.

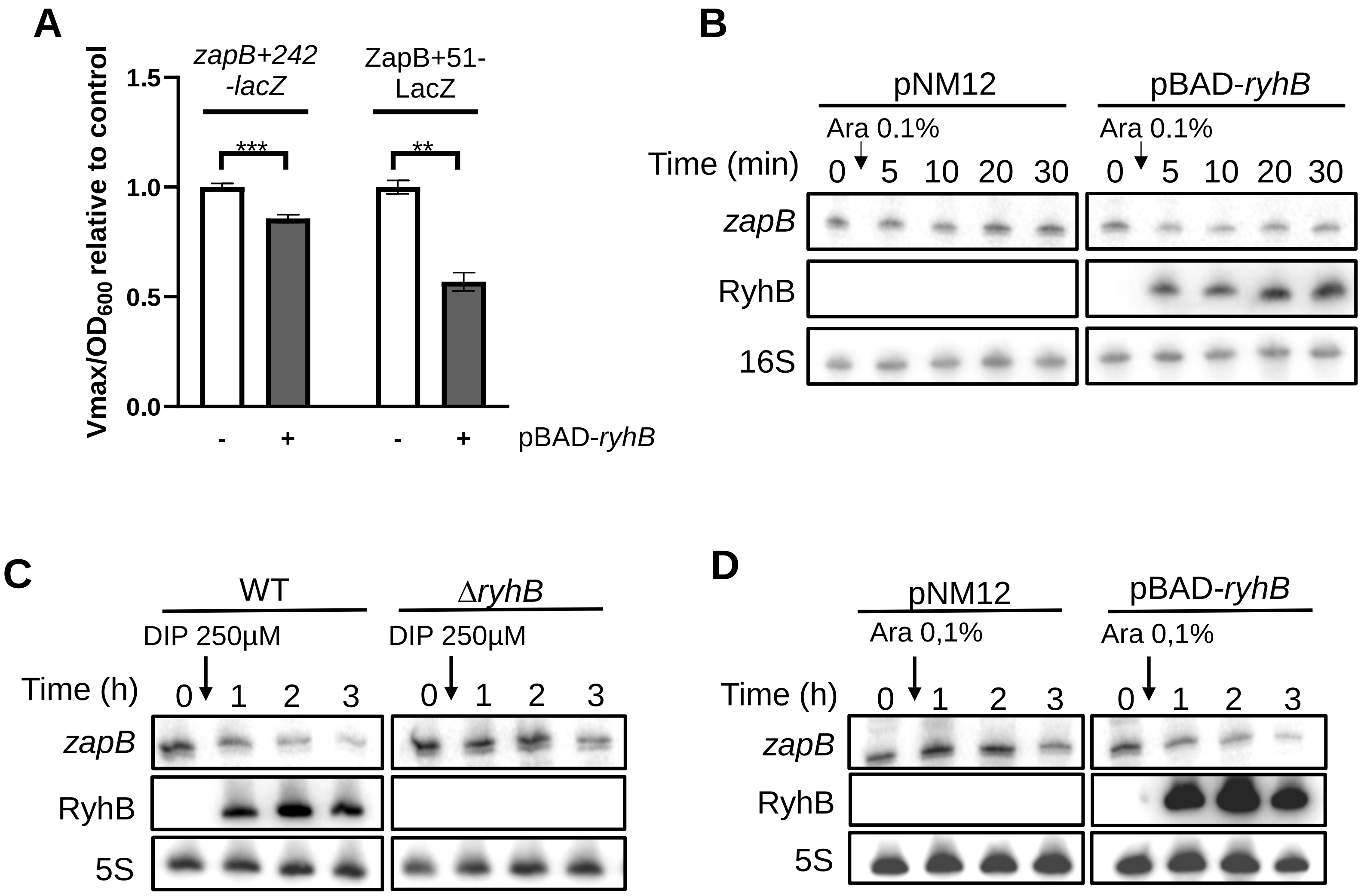

Figure S2. RyhB overexpression does not induce rapid degradation of *zapB* mRNA. A) β-galactosidase assays using transcriptional *zapB+242-lacZ* or translational ZapB+51-LacZ fusions in LB media. RyhB expression from pBAD promoter (Δ*ryhB* background) was induced by addition of 0.1% arabinose (Ara) at OD_{600nm}=0.1. Samples were taken at an OD_{600nm}=1.5. pNM12 plasmid was used as a control. Data represents two independent experiments. Student t-test with Welch's correction (**p=0.0070 and ***p=0.0005). **B)** Northern blot of *zapB* mRNA. RyhB expression from pBAD promoter (Δ*ryhB* background) was induced by addition of 0.1% arabinose (Ara) at OD_{600nm}=0.5. pNM12 plasmid was used as a control. 16S rRNA was used as a loading control. Data are representative of three independent experiments. **C)** Northern blot of *zapB* mRNA and RyhB following addition of 250µM 2,2′-dipyridyl (DIP) at OD_{600nm}=0.1 in WT or Δ*ryhB* background. 16S rRNA was used as a loading control. Data are representative of three independent experiments. **D)** Northern blot of *zapB* mRNA. RyhB expression from pBAD promoter (Δ*ryhB* background) was induced by addition of 0.1% arabinose (Ara) at OD_{600nm}=0.5. pNM12 plasmid was used as a control. 16S rRNA was used as a loading control. Data are representative of three independent experiments.

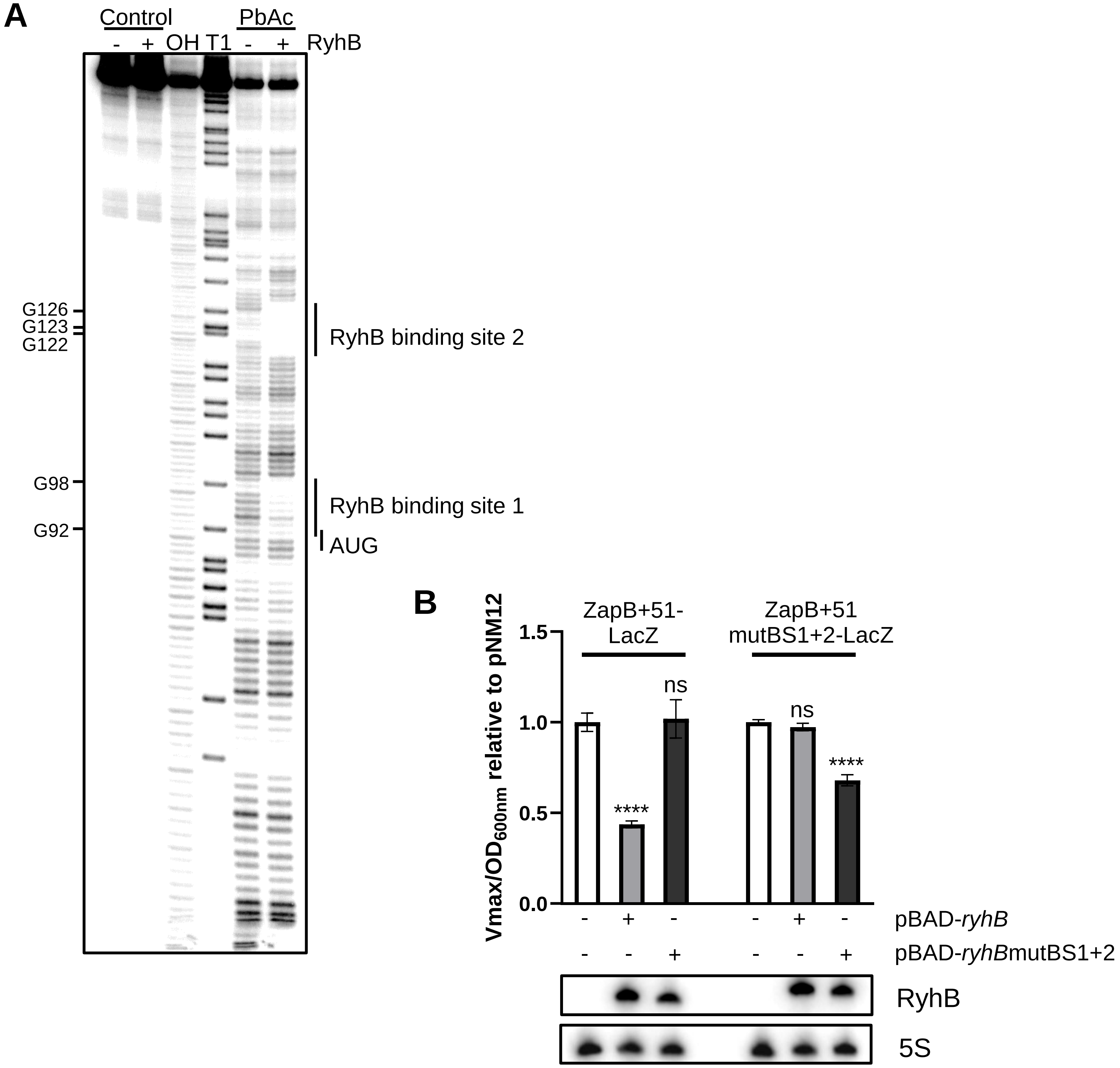

Figure S3. Each RyhB binding sites on *zapB* mRNA act independently from each other. A) Lead acetate (PbAc) probing assay of radiolabelled *zapB*, in the presence or absence of RyhB. OH: alkaline ladder, T1: RNase T1 ladder. **B)** β-galactosidase assays using the ZapB+51-LacZ and ZapB+51mutBS1+2-LacZ translational fusions in Δ*ryhB* background. Expression of RyhB and RyhBmutBS1+2 from pBAD promoter was induced by addition of 0.1% arabinose at OD_{600nm}=0.1. Samples were taken at OD_{600nm}=1.5. pNM12 plasmid was used as a control. Northern blot assays were performed at the same time to monitor levels of RyhB sRNA. 5S rRNA was used as a loading control. Data represents three independent experiments. Two-way ANOVA with Dunnett's multiple comparisons test using pNM12 as control (****p<0.0001).

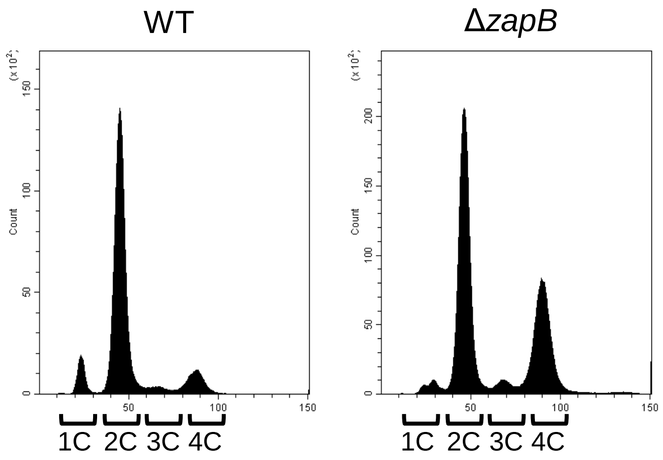

Figure S5. Controls cells (WT and Δ*zapB* backgrounds) used to determine chromosome distribution for flow cytometry assays. Cephalexin and rifampicin were used to inhibit cell growth and stop transcription respectively. 1C: 1 chromosome in a cell, 2C: 2 chromosomes in a cell, 3C: 3 chromosomes in a cell and 4C: 4 chromosomes in a cell.

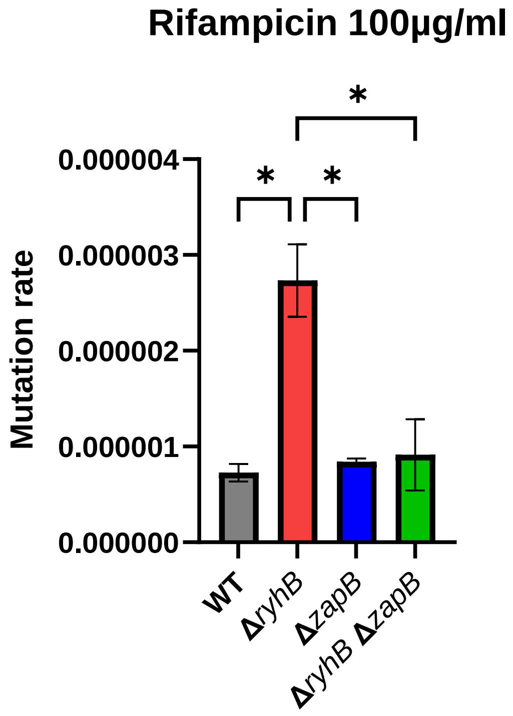

Figure S6. Absence of RyhB affects DNA replication fidelity. Mutation rate in WT, Δ*ryhB*, Δ*zapB* and Δ*ryhB* Δ*zapB* strains grown in M63 minimal media and plated on LB agar + 100µg/ml rifampicin. Mutation rate is reported as CFU on LB-rifampicin plates/total CFU. Data represents three independent experiments. One-way ANOVA with Dunnett's multiple comparisons test (*p between 0.0172 and 0.0396).
